# Clinical Value of Lymph Node Ratio Integration with the 8^th^ Edition of the UICC TNM Classification and 2015 ATA Risk Stratification Systems for Recurrence Prediction in Papillary Thyroid Cancer

**DOI:** 10.1038/s41598-019-50069-4

**Published:** 2019-09-16

**Authors:** Jandee Lee, Seul Gi Lee, Kwangsoon Kim, Seung Hyuk Yim, Haengrang Ryu, Cho Rok Lee, Sang Wook Kang, Jong Ju Jeong, Kee-Hyun Nam, Woong Youn Chung, Young Suk Jo

**Affiliations:** 1 Department of Surgery, Open NBI Convergence Technology Research Laboratory, Severance Hospital, Yonsei Cancer Center, Yonsei University College of Medicine, Seoul, South Korea; 2 Department of Surgery, Eulji University School of Medicine, Daejeon, South Korea; 3 Department of Surgery, Hongik Hospital, Seoul, South Korea; 4 Brain Korea 21 PLUS Project for Medical Science, Yonsei University, Seoul, South Korea; 5 Department of Internal Medicine, Severance Hospital, Yonsei Cancer Center, Yonsei University College of Medicine, Seoul, South Korea

**Keywords:** Thyroid cancer, Surgical oncology

## Abstract

Recently, the 2015 American Thyroid Association (ATA) risk stratification and the 8^th^ edition of the American Joint Committee on Cancer/Union for International Cancer Control (AJCC/UICC) TNM staging system were released. This study was conducted to assess the clinical value of the lymph node ratio (LNR) as a predictor of recurrence when integrated with these newly released stratification systems, and to compare the predictive accuracy of the modified systems with that of the newly released systems. The optimal LNR threshold value for predicting papillary thyroid cancer (PTC) recurrence was 0.17857 using the Contal and O’Quigley method. The 8^th^ edition of the AJCC/UICC TNM staging system with the LNR and the 2015 ATA risk stratification system with the LNR were significant predictors of recurrence. Furthermore, calculation of the proportion of variance explained (PVE), the Akaike information criterion (AIC), Harrell’s c index, and the incremental area under the curve (iAUC) revealed that the 8^th^ edition of the TNM staging system with the LNR, and the 2015 ATA risk stratification system with the LNR, showed the best predictive performance. Integration of the LNR with the TNM staging and the ATA risk stratification systems should improve prediction of recurrence in patients with PTC.

## Introduction

Thyroid cancer is the most common endocrine cancer. Since the incidence of thyroid cancer is increasing, several risk stratification and treatment guidelines for patients with differentiated thyroid cancer (DTC) have been devised^1–6^. Recently, to provide a more accurate system that predicts disease-specific survival (DSS), the 8^th^ edition of the TNM staging system was released by the American Joint Committee on Cancer/Union for International Cancer Control (AJCC/UICC). However, the 8^th^ TNM staging system has a limitation in terms of estimating recurrence risk and reflecting the biological behavior of DTC^7–9^. In practice, predicting recurrence might be more important than predicting DSS since DTC has a much lower mortality rate than other common malignancies.

The 8^th^ TNM staging system underestimates the significance of lymph node metastasis. By contrast, the revised 2015 ATA guidelines point out the importance of metastatic lymph nodes (MLNs), indicating that the number and size of MLNs in the neck are predictors of papillary thyroid cancer (PTC) recurrence. In line with this, complete evaluation of MLNs is now conducted more accurately. However, this new system also has a limitation in that the stratification strategy, which comprises three groups, is too simple for a personalized medicine approach that needs to reflect the diverse clinical or pathological behaviors of DTC^10–15^.

The ratio of MLNs to examined LNs (the lymph node ratio [LNR]) has also been investigated as an important predictor of PTC recurrence (i.e., recurrence-free survival [RFS]) and DSS^16,17^. Here, we examined clinico-pathological data in a cohort of PTC patients who underwent total thyroidectomy (TT) and central compartment node dissection (CCND), with or without modified radical neck dissection (MRND), at a single tertiary referral hospital. To determine the optimal LNR threshold for PTC recurrence, we performed statistical analyses using the Contal and O’Quigley method based on the log rank test. We then examined the effect of integrating the LNR with the 8^th^ TNM staging or 2015 ATA risk stratification system to predict RFS. Finally, we compared the predictive accuracy of this modified system with that of the previous/current TNM and ATA risk stratification systems.

## Results

### Baseline clinico-pathological data of the study patients

As shown in Table 1, the mean age of the patients was 45.0 ± 12.3 years (range, 8–83 years) and 2096 (86.5%) were female. The mean tumor size was 1.37 ± 1.04 cm, and the multiplicity and bilaterality of cancer were 1087 (44.8%) and 813 (33.5%), respectively. Overall, 1651 (68.1%) patients had pathologically confirmed extrathyroidal extension (ETE), with 1229 (50.6%) having minimal ETE. The majority (95.4%) received postoperative radioactive iodine (RAI) ablation. The mean number of retrieved and metastatic LNs was 9.0 ± 4.4 and 2.7 ± 3.4, respectively, in the central compartment and 28.7 ± 16.9 and 5.2 ± 5.2, respectively, in the lateral compartment.

**Table 1:** Baseline clinico-pathological characteristics of the study patients.

	Total 2424 patients
Age (years)	45.0 ± 12.3 (8–83)
M:F	1:6.4 (328:2096)
Tumor size (cm)	1.37 ± 1.04
Multiplicity	1087 (44.8%)
Bilaterality	813 (33.5%)
Extrathyroidal extension	1651 (68.1%)
Minimal	1229 (50.6%)
Gross	422 (17.5%)
Surgery	
TT with CCND	1422 (58.7%)
TT with CCND + MRND	1002 (41.3%)
Postoperative RAI ablation	2313 (95.4%)
Low/high dose	1653 (71.5%)/660 (28.5%)
Metastatic/retrieval LNs in central compartment	2.7 ± 3.4/9.0 ± 4.4
Metastatic/retrieval LNs in lateral compartment	5.2 ± 5.2/28.7 ± 16.9
Mean follow-up duration (months)	114.0 ± 36.1 (range, 63–265)

Abbreviations: TT, total thyroidectomy; CCND, central compartment node dissection; MRND, modified radical neck dissection; RAI, radioactive iodine; LNs, lymph nodes.

### Classification of patients with PTC according to the 8^th^ edition of the AJCC/UICC staging system combined with the LNR

The 8^th^ TNM staging system recommends that the age threshold should be changed from 45 years to 55 years. Remarkably, minor ETE was removed, resulting in new categories T3a and T3b. The definition of a central neck LN (N1a) was also changed to include both level VI and level VII compartments^8,15,18^. However, here, there was no change in the N stage because no patient had level VII LN metastasis. Supplementary Table 1 shows patients classified according to the 7^th^ and 8^th^ TNM staging systems. As expected, a significant number of T3 patients from the 7^th^ T staging system were reclassified as T1 (1074, 44.3%) and T2 (155, 6.4%) in the 8^th^ T staging. Accordingly, a large number of stage II, III, and IV patients from the 7^th^ TNM were reclassified as stage I (724, 29.9%) and II (279, 11.5%).

As described in Methods, the optimal threshold value calculated for the LNR was 0.17857. Combining this value with the 8^th^ TNM staging, we classified patients into eight new groups: stage I (low LNR, 1189 [49.0%]), stage I (high LNR, 882 [36.4%]), stage II (low LNR, 128 [5.3%]), stage II (high LNR, 162 [6.7%]), stage III (low LNR, 19 [0.8%]), stage III (high LNR, 35 [1.4%]), stage IV (low LNR, 6 [0.2%]), and stage IV (high LNR, 3 [0.1%]) (Supplementary Table 2).

### Classification of patients with PTC according to the 2015 ATA risk stratification system combined with the LNR

As shown in Supplementary Table 3, the 2009 ATA risk stratification system identified 377 (15.6%), 1910 (78.8%), and 137 (5.7%) patients as low-, intermediate-, or high-risk, respectively. In the 2015 ATA risk stratification system, the number of patients in each group was 630 (26.0%), 1651 (68.1%), and 143 (5.9%), respectively. Using the same LNR threshold (0.17857), patients were reclassified into six new groups: low-risk (535 with a low LNR [22.1%] and 95 with a high LNR [3.9%]), intermediate-risk (754 with a low LNR [31.1%] and 897 with a high LNR [37.0%]), and high-risk (53 with a low LNR [2.2%] and 90 with a high LNR [3.7%]).

### Risk factors for tumor recurrence

Recurrence was detected in 134 (5.5%) patients during follow-up, and the mean disease-free survival time was 46.2 months (range, 12–170 months). As presented in Supplementary Table 4, diverse clinico-pathological factors as well as the mean LNR differed between the recurrence and non-recurrence groups (mean values, 0.35 *vs*. 0.13, respectively; *P* < 0.0001). Combining the LNR with the 8^th^ TNM staging and 2015 ATA stratification systems revealed that the recurrence group tended to have a higher LNR than the non-recurrence group, particularly the 8^th^ TNM stage II and III and the 2015 ATA intermediate- and high-risk groups.

Univariate and multivariate analyses performed to estimate the hazard ratio (HR) of clinico-pathological factors revealed that advanced stage, especially 8^th^ TNM III and IV, showed an increasing HR for PTC recurrence (Table 2). Consistent with the results from Supplementary Table 4, univariate analysis revealed that a high LNR increased the HR for 8^th^ TNM stages I, II, and III, and multivariate analysis revealed an increase for all stages. Univariate and multivariate analyses conducted to calculate the HR of clinico-pathological factors in the ATA risk stratification systems also revealed an association between advanced risk and a higher HR (Table 3). Again, when combined with the LNR, univariate and multivariate analyses revealed that a high LNR increased the HR for all risk groups.

**Table 2:** Univariate and multivariate analyses of baseline variables for recurrence-free survival with the TNM staging system.

	Univariate		Multivariate	
	HR (95% CI)	*P*-value	HR (95% CI)	*P*-value
**8** ^**th**^ **TNM**				
I	ref.		ref.	
II	1.373 (0.842–2.239)	0.2042	1.227 (0.751–2.006)	0.4142
III	5.727 (3.226–10.165)	<0.0001	3.300 (1.841–5.917)	<0.0001
IVA	17.386 (3.401–88.875)	0.0006	20.499 (3.746–112.179)	0.0005
IVB	1.436 (0.087–23.711)	0.8005	2.562 (0.154–42.704)	0.5123
**8** ^**th**^ **TNM with LNR**				
I with low LNR	ref.		ref.	
I with high LNR	6.736 (3.995–11.358)	<0.0001	4.925 (2.896–8.375)	<0.0001
II with low LNR	3.501 (1.404–8.73)	0.0072	3.102 (1.241–7.753)	0.0154
II with high LNR	5.752 (2.798–11.825)	<0.0001	4.727 (2.291–9.755)	<0.0001
III with low LNR	9.247 (2.409–35.491)	0.0012	6.974 (1.804–26.954)	0.0049
III with high LNR	26.975 (12.668–57.44)	<0.0001	14.753 (6.803–31.994)	<0.0001
IV with low LNR	17.491 (3.219–95.042)	0.0009	12.911 (2.356–70.740)	0.0032
IV with high LNR	11.693 (0.674–202.781)	0.0912	18.028 (1.023–317.820)	0.0482

Low LNR indicates <0.17857, and high LNR indicates ≥0.17857.

Abbreviations: ETE, extrathyroidal extension; LNR, lymph node ratio.

**Table 3:** Univariate and multivariate analyses of baseline variables for recurrence-free survival with ATA risk stratification.

	Univariate		Multivariate	
	HR (95% CI)	*P*-value	HR (95% CI)	*P*-value
**2015 ATA**				
Low	ref.		ref.	
Intermediate	7.2 (3.166–16.374)	<0.0001	3.396 (1.454–7.933)	0.0047
High	14.006 (5.56–35.284)	<0.0001	5.328 (2.052–13.834)	0.0006
**2015 ATA with LNR**				
Low with low LNR	ref.		ref.	
Low with high LNR	2.815 (0.516–15.37)	0.232	2.671 (0.489–14.584)	0.2567
Intermediate with low LNR	3.236 (1.095–9.562)	0.0336	3.009 (1.018–8.896)	0.0464
Intermediate with high LNR	14.308 (5.258–38.933)	<0.0001	10.011 (3.657–27.404)	<0.0001
High with low LNR	10.239 (2.561–40.939)	0.001	7.549 (1.88–30.318)	0.0044
High with high LNR	22.668 (7.462–68.866)	<0.0001	14.129 (4.604–43.359)	<0.0001

Low LNR indicates <0.17857, and high LNR indicates ≥0.17857.

Abbreviations: ETE, extrathyroidal extension; LNR, lymph node ratio.

### Comparison of performance and predictive accuracy of the TNM staging and ATA risk stratification systems for RFS when combined with the LNR

To compare the predictive power of the TNM staging systems with or without the LNR, we calculated the PVE value, the AIC, Harrell’s c index, and the iAUC using the 7^th^ TNM, 8^th^ TNM, and 8^th^ TNM with the LNR (threshold = 0.4), and 8^th^ TNM with the LNR (threshold = 0.17857). Since our own and other previous studies suggested an optimal LNR threshold of 0.4 for predicting recurrence or disease-specific mortality, we decided to use 0.4 as the second LNR threshold in the analysis^16,19^. The 8^th^ TNM with the LNR (threshold = 0.17857) showed the highest predictive accuracy of all four calculation methods (Table 4). Consistently, among the 2009 ATA, 2015 ATA, 2015 ATA with the LNR (threshold = 0.4), and 2015 ATA with the LNR (threshold = 0.17857), the 2015 ATA with the LNR (threshold = 0.17857) showed the greatest predictive accuracy (Table 5).

**Table 4:** Comparative analysis of the performance and predictive accuracy of the TNM staging system for recurrence-free survival.

	PVE	AIC	Harrell’s c index (95% CI)	iAUC (95% CI)
7^th^ TNM	1.577	2033.548	0.6375 (0.5939–0.6802)	0.6371 (0.5951–0.6780)
8^th^ TNM	1.082	2043.721	0.5654 (0.5317–0.6054)	0.5648 (0.5322–0.6050)
8^th^ TNM (LNR threshold = 0.4)	2.518	2014.293	0.6704 (0.6268–0.7129)	0.6637 (0.6193–0.7047)
8^th^ TNM (LNR threshold = 0.17857)	4.127	1973.947	0.7282 (0.6946–0.7588)	0.7203 (0.6869–0.7506)

Abbreviations: PVE, proportion of variance explained; AIC, Akaike information criterion; iAUC, incremental area under the curve; LNR, lymph node ratio.

**Table 5:** Comparative analysis of the performance and predictive accuracy of the ATA risk stratification for recurrence-free survival.

	PVE	AIC	Harrell’s c index (95% CI)	iAUC (95% CI)
2009 ATA	1.273	2035.01	0.5879 (0.5599–0.6185)	0.5914 (0.5627–0.6223)
2015 ATA	2.117	2014.194	0.6296 (0.5998–0.6597)	0.6322 (0.6019–0.6615)
2015 ATA (LNR threshold = 0.4)	3.264	1991.645	0.7009 (0.6654–0.7362)	0.7007 (0.6662–0.7355)
2015 ATA (LNR threshold = 0.17857)	4.026	1972.462	0.7262 (0.6931–0.7573)	0.7225 (0.6899–0.7531)

Abbreviations: PVE, proportion of variance explained; AIC, Akaike information criterion; iAUC, incremental area under the curve; LNR, lymph node ratio.

## Discussion

This study aimed to estimate the effect of integrating the LNR with TNM staging and ATA risk stratification systems to predict PTC recurrence. Indeed, the 8^th^ TNM staging system is important to assess the risk of mortality, and may not be appropriate to predict the risk for disease recurrence. Although the revised 2015 ATA guidelines emphasized the characteristics of MLNs including the number of involved LNs, the size of the largest involved LN, and extranodal extension, these potential prognostic factors do not have pertinent threshold values^18,20^. To optimize management, more tailored risk stratification of LN status such as the LNR is needed in PTC patients to provide an objective determination of prognosis for these patients.

In fact, the LNR has been known as a prognostic variable in PTC. To inform the management of low- and high-risk tumors, surgeons should ensure that the LN yield is adequate during surgery and pathologists also need to perform a careful histologic examination of specimens. Based on this idea and on the relatively large amount of existing research data, we postulated that, among the diverse characteristics of MLNs, the LNR was the most suitable for clinical application^19,21–28^. Supporting our postulation, a higher LNR increased the HR of same-stage tumors, even after adjusting for epidemiological and basic clinico-pathological tumor characteristics. For TNM stage I, a high LNR increased the HR to 4.925 (confidence interval [CI], 2.896–8.375), indicating that the LNR might be an important predictor of recurrence, even for low-stage disease. Consistent with this, for stage II a high LNR increased the HR from 3.102 (CI, 1.241–7.753) to 4.727 (CI, 2.291–9.755). These results for stage I and II PTC suggest that a high LNR is the most powerful predictor of recurrence in those with low-stage disease since the HRs for stage I and II PTC are quite similar (4.925 *vs*. 4.727, respectively). Further, integrating the LNR with the 2015 ATA risk stratification system enhanced the predictive performance. The HR of the intermediate-risk group with a high LNR was higher than that of the high-risk group with a low LNR (10.011; CI, 3.657–27.404 *vs*. 7.549; CI, 1.88–30.318).

The problem with the LNR as a predictor is determining an optimal threshold since the definition of the LNR and the extent of surgery differ between previous studies. In fact, in a previous study, we reported an optimal threshold of 0.4 or 0.5 for central and lateral compartment LN groups, respectively^16^. We did not include patients with no MLNs (pN0) in the previous study, and LNR thresholds were calculated by selecting the inflection points of the binomial logistic regression curves for the probability of recurrence. However, in the present study, we included pN0 patients to discover the appropriate LNR across all TNM stages. We also re-assessed the optimal threshold using the Contal and O’Quigley method, a technique that uses the log rank test and is suitable for determining thresholds across all stages. This re-assessment yielded a threshold of 0.17857.

To compare the two thresholds, we estimated the performance of the TNM staging and ATA risk stratification systems using four statistical calculations: PVE, AIC, Harrell’s c index, and iAUC. According to the results, the 8^th^ TNM staging system plus a LNR of 0.17857 was the most accurate predictor of RFS and DSS (data not shown). Harrell’s c index and iAUC could not be calculated for DSS due to the low mortality rate in this cohort. Likewise, all four calculation methods indicated that the 2015 ATA risk stratification system with a LNR of 0.17857 was the most accurate predictor of RFS and DSS (data not shown). In terms of predicting overall survival (OS), the 2015 ATA risk stratification yielded a higher PVE (1.174) when integrated with the previous LNR of 0.4 than when integrated with the present LNR of 0.17857 (PVE = 1.165) (data not shown). Taken together, these statistical analyses suggested that the new LNR of 0.17857 was useful as a standard indicator to determine RFS across all stages of PTC, and the previous LNR of 0.4 was also valuable to predict OS in patients with pN1. Therefore, our data show the critical importance of the LNR threshold if the LNR is to be used as a prognostic marker.

This study has a few limitations. First, it was retrospective and we could not verify the precise pathologic status of MLNs including the size of micrometastatic foci and extranodal extensions. Second, the study population was sampled from a single tertiary referral hospital and might thus have been biased toward more advanced disease cases. Third, the study included only patients who underwent TT with CCND or MRND (other surgical procedures such as hemi-thyroidectomy or lobectomy were excluded). These inclusion and exclusion criteria might introduce selection biases when determining the LNR threshold. Finally, it was impossible to perform head-to-head comparisons of the TNM staging and ATA risk stratification systems in terms of OS, RFS, and DSS since statistical differences in covariates between the two systems limited scientific analyses.

In conclusion, integration of the LNR with the TNM staging and the ATA risk stratification systems increases the accuracy of predicting PTC recurrence. Further studies will be undertaken to examine the predictive role of well-defined analyses of MLNs in a clinical setting.

## Methods

### Patients

The clinico-pathologic characteristics of 2705 patients with PTC (from January 1991 to December 2010) were analyzed retrospectively via complete review of medical charts and pathology reports. To ensure adequate LN dissection yield, patients with fewer than six central LNs harvested by CCND or fewer than 18 LNs harvested by MRND were excluded (n = 281). Among the patients finally enrolled (n = 2424), 1422 (58.7%) underwent TT with prophylactic or therapeutic CCND, and 1002 (41.3%) underwent TT with prophylactic or therapeutic CCND, with therapeutic MRND performed for clinically suspicious or pathologically confirmed N1b nodes. The mean follow-up duration was 114.0 months (range, 63–265 months). Of the patients who underwent TT, 2313 (95.4%) received RAI ablation at 4–8 weeks post-surgery using a dose based on the ATA guidelines. The follow-up protocol was also based on ATA guidelines. PTC recurrence was confirmed by imaging modalities and/or pathologic diagnosis by ultrasound-guided fine needle aspiration biopsy (US-FNAB). Some patients were included in previous analyses published in prior papers^16,29^.

### Measurement of the LNR

Two experienced pathologists re-examined (independently) the LN status of specimens for evidence of LN metastasis. The LNR was defined as the number of MLNs divided by the total number of LNs retrieved from the central compartment with or without nodes from lateral compartments. The Contal and O’Quigley method for PTC recurrence was used to calculate the threshold value for the LNR; the value was 0.17857^30^.

### Statistical analysis

Student’s t-test and the Chi-square test or Wilcoxon rank sum test were used to compare groups as appropriate. Univariate and multivariate Cox regression analyses were performed to identify independent predictors of RFS. *P* < 0.05 indicated statistical significance. To estimate the performance of the TNM staging and ATA risk stratification systems, four statistical parameters were calculated: the proportion of variance explained (PVE), the Akaike information criterion (AIC), Harrell’s c index, and the time-dependent receiver operating characteristics (ROC) curve (incremental area under the curve, iAUC). All statistical analyses were performed using IBM SPSS statistics 23.0 (SPSS Inc., Chicago, IL, USA), SAS (version 9.4, SAS Inc., Cary, NC, USA) and R package version 3.1.3 (http://www.R-project.org).

### Ethical approval and informed consent

This study was approved by the institutional review board of Severance Hospital and was conducted in accordance with the recommendations of the institutional review board, which waived the requirement for informed consent due to its retrospective nature.

## Supplementary information

Supplementary Information

## Data Availability

The datasets generated during and/or analysed during the current study are available from the corresponding author on reasonable request.
