# Supplementary material for: Clinical Value of Lymph Node Ratio Integration with the 8th Edition of the UICC TNM Classification and 2015 ATA Risk Stratification Systems for Recurrence Prediction in Papillary Thyroid Cancer
Source: Sci Rep. 2019 Sep 16;9:13361. doi: 10.1038/s41598-019-50069-4 (PMC6746784; doi:10.1038/s41598-019-50069-4)
Supplement: Supplementary file 1 — Supplementary Information [file 41598_2019_50069_MOESM1_ESM.pdf]

Supplementary Information

Clinical Value of Lymph Node Ratio Integration With the 8th Edition of the UICC TNM Classification and 2015 ATA Risk Stratification Systems for Recurrence Prediction in Papillary Thyroid Cancer

Jandee Lee[1*], Seul Gi Lee[2*], Kwangsoon Kim[1], Seung Hyuk Yim[1], Haengrang Ryu[3], Cho Rok Lee[1], Sang Wook Kang[1], Jong Ju Jeong[1], Kee-Hyun Nam[1], Woong Youn Chung[1], and Young Suk Jo[4,5]

[1]Department of Surgery, Open NBI Convergence Technology Research Laboratory, Severance Hospital, Yonsei Cancer Center, Yonsei University College of Medicine, Seoul, South Korea
[2]Department of Surgery, Eulji University School of Medicine, Daejeon, South Korea
[3]Department of Surgery, Hongik Hospital, Seoul, South Korea
[4]Brain Korea 21 PLUS Project for Medical Science, Yonsei University, Seoul, South Korea
[5]Department of Internal Medicine, Severance Hospital, Yonsei Cancer Center, Yonsei University College of Medicine, Seoul, South Korea

[*]These two authors equally contributed to this work.

Corresponding author
Young Suk Jo, MD, PhD.
Associate Professor
Department of Internal Medicine, Yonsei University College of Medicine, 120-752, Seoul, Korea
Tel: +82-2-2228-0888; Fax: +82-2-393-6884; E-mail: joys@yuhs.ac

This file includes four Supplementary Tables.

Supplementary Table 1. T stage and TNM stage migration of patients according to 7th TNM stage and 8th TNM stage

A. T stage

		8th TNM				
		T1 (n=1782)	T2 (n=220)	T3 (n=243)	T4a (n=172)	T4b (n=7)
7th TNM	T1 (n=708)	708	-	-	-	
	T2 (n=65)	-	65	-	-	
	T3 (n=1472)	1074	155	243	-	
	T4a (n=172)	-	-	-	172	
	T4b (n=7)					7

B. TNM stage

		8th TNM			
		I (n=2071)	II (n=290)	III (n=54)	IV (n=9)
7th TNM	I (n=1344)	1344	-	-	-
	II (n=14)	3	11	-	-
	III (n=639)	501	138	-	-
	IV (n=427)	223	141	54	9

Supplementary Table 2. Assessment of TNM staging integrated with LNR in patients based on 7th and 8th TNM staging systems

	7th TNM (n=2424)	8th TNM (n=2424)
I	1344 (55.4)	2071 (85.4)
Low LNR		1189 (49.0)
High LNR		882 (36.4)
II	14 (0.6)	290 (12.0)
Low LNR		128 (5.3)
High LNR		162 (6.7)
III	639 (26.4)	54 (2.2)
Low LNR		19 (0.8)
High LNR		35 (1.4)
IVA/IVB/IVC	411 (17.0)/5 (0.2)/11 (0.5)	2 (0.1)/7 (0.3)/-
Low LNR		6 (0.2)
High LNR		3 (0.1)

Data are expressed as patient number (%).
Low LNR indicates <0.17857, and high LNR indicates ≥0.17857.

Supplementary Table 3. Assessment of risk stratification integrated with LNR in patients based on the 2009 and 2015 ATA guidelines

	2009 ATA guideline (n=2424)	2015 ATA guideline (n=2424)
Low risk	377 (15.6)	630 (26.0)
Low LNR		535 (22.1)
High LNR		95 (3.9)
Intermediate risk	1910 (78.8)	1651 (68.1)
Low LNR		754 (31.1)
High LNR		897 (37.0)
High risk	137 (5.7)	143 (5.9)
Low LNR		53 (2.2)
High LNR		90 (3.7)

Data are expressed as patient number (%).
Low LNR indicates <0.17857, and high LNR indicates ≥0.17857.

Supplementary Table 4. Clinico-pathological features according to recurrence in patients with papillary thyroid cancer

	Recurrence (n=2424)		
	No (n=2290, 94.47%)	Yes (n=134, 5.53%)	p-value
Age (years)	45 (36–53)	43.5 (31–54)	0.1288
Sex (male)	291 (12.71%)	37 (27.61%)	<0.0001
Tumor size (cm)	1.1 (0.7–1.6)	1.95 (1.2–2.8)	<0.0001
Multiplicity (yes)	1009 (44.06%)	78 (58.21%)	0.0014
Bilaterality (yes)	750 (32.75%)	63 (47.02%)	0.0007
Extrathyroidal extension (yes)	1522 (66.46%)	116 (86.57%)	<0.0001
LNR	0.13 (0–0.31)	0.35 (0.21–0.53)	<0.0001
LNR two group (high)	974 (42.53%)	108 (80.6%)	<0.0001
Adjuvant RAI Tx 30/100/150/≥200 mCi	107 (4.68%)/ 1576 (68.85%)/ 517 (22.59%)/ 89 (3.89%)	4 (2.99%)/ 77 (57.46%)/ 42 (31.34%)/ 11 (8.21%)	0.0050
7th TNM			<0.0001
I	1277 (55.76%)	67 (50%)	
II	13 (0.57%)	1 (0.75%)	
III	622 (27.16%)	17 (12.69%)	
IVA	363 (15.85%)	48 (35.82%)	
IVB	4 (0.18%)	1 (0.75%)	
IVC	11 (0.48%)	0 (0)	
8th TNM			<0.0001
I	1970 (86.03%)	101 (75.37%)	
II	271 (11.83%)	19 (14.18%)	
III	41 (1.79%)	13 (9.7%)	
IVA	1 (0.04%)	1 (0.75%)	
IVB	7 (0.31%)	0 (0)	
8th TNM with LNR			<0.0001
I with low LNR	1172 (51.18%)	17 (12.69%)	
I with high LNR	798 (34.85%)	84 (62.69%)	
II with low LNR	122 (5.33%)	6 (4.48%)	
II with high LNR	149 (6.51%)	13 (9.70%)	
III with low LNR	17 (0.74%)	2 (1.49%)	
III with high LNR	24 (1.05%)	11 (8.21%)	
IV with low LNR	5 (0.22%)	1 (0.75%)	
IV with high LNR	3 (0.13%)	0 (0)	
2009 ATA			<0.0001
Low	373 (16.29%)	4 (2.99%)	
Intermediate	1797 (78.47%)	113 (84.33%)	
High	120 (5.24%)	17 (12.69%)	
2015 ATA			<0.0001
Low	624 (27.25%)	6 (4.48%)	
Intermediate	1541 (67.29%)	110 (82.09%)	
High	125 (5.46%)	18 (13.43%)	
2015 ATA with LNR			<0.0001
Low with low LNR	531 (23.19%)	4 (2.99%)	
Low with high LNR	93 (4.06%)	2 (1.49%)	
Intermediate with low LNR	736 (32.14%)	18 (13.43%)	
Intermediate with high LNR	805 (35.15%)	92 (68.66%)	
High with low LNR	49 (2.14%)	4 (2.99%)	
High with high LNR	76 (3.32%)	14 (10.45%)	

Low LNR indicates <0.17857, and high LNR indicates ≥0.17857.
